# Performance of Recycled Polylactic Acid/Amorphous Polyhydroxyalkanoate Blends

**DOI:** 10.3390/polym16091230

**Published:** 2024-04-28

**Authors:** Simran Chatrath, Mansour Alotaibi, Carol Forance Barry

**Affiliations:** Department of Plastics Engineering, University of Massachusetts Lowell, Lowell, MA 01879, USA

**Keywords:** polylactic acid, amorphous polyhydroxyalkanoate, biodegradable blends

## Abstract

Blends of polylactic acid (PLA) with amorphous polyhydroxyalkanoate (aPHA) are less brittle than neat PLA, thus enabling their use as biodegradable packaging. This work investigated the impact of recycling on the properties of neat PLA and PLA/aPHA blends with 90 and 75 wt. % PLA. After the materials were subjected to five heat histories in a single-screw extruder, the mechanical, rheological, and thermal properties were measured. All recycled compounds with 100% PLA and 75% PLA had similar decomposition behavior, whereas the decomposition temperatures for the blends with 90% PLA decreased with each additional heat cycle. The glass transition and melting temperatures were not impacted by reprocessing, but the crystallinity increased with more heat cycles. The complex viscosity of the reprocessed PLA and PLA/aPHA blends was much lower than for the neat PLA and increasing the number of heat cycles produced smaller reductions in the complex viscosity of 100% PLA and the blend with 90% PLA; no change in complex viscosity was observed for blends with 75% PLA exposed to 2 to 5 heat cycles. The tensile properties were not affected by reprocessing, whereas the impact strength for the 75% PLA blend decreased with reprocessing. These properties suggest that users will be able to incorporate scrap into the neat resin for thermoformed packaging.

## 1. Introduction

Polymers commonly used for packaging include polyethylene (PE), polypropylene (PP), and polyethylene terephthalate (PET) [1]. The packaging often is manufactured using thermoforming, specifically roll-to-roll thermoforming. The market for thermoformed plastics market is growing and is expected to reach USD 20.68 billion in 2030 [2]. During roll-to-roll thermoforming, the containers are formed from extruded sheet, creating scrap levels as high as 60% [3,4]. Recent developments in roll-to-roll thermoforming have focused on better part layout to reduce scrap levels [3], in-house recycling of the polymers, and better systems for collecting and processing post-consumer recycle (PCR).

When looking at alternatives for fossil fuel-based PE, PP, and PET, there are three options: (1) bio-based polymers, (2) biodegradable polymers, and (3) bio-based, biodegradable polymers. Bio-based polymers are either derived or partly derived from biomass (plants) and materials such as fats and vegetable oils, gluten [5], egg white protein [6], and starch [7,8]. Currently available bio-based plastics include bio-polyethylene (bio-PE), bio-polyethylene terephthalate (bio-PET) [9], and polytrimethylene terephthalate (PTT) [10]; there also are blends of polymers with starch and thermoplastic starch (TPS) [9]. These bio-based polymers, however, are not biodegradable [11] and they must be recycled. Biodegradable polymers made from fossil fuels are a second option. These polymers can undergo a biochemical process in which microorganisms available in the environment convert materials into water, carbon dioxide, and biomass. The biodegradation process is affected by factors such as polymer morphology, structure, chemical treatment, and molecular weight [12]. Polymers such as polybutylene succinate (PBS), polycaprolactone (PCL), and polybutylene adipate-co-terephthalate (PBAT) have been explored for applications in packaging [13]. PBAT, which has properties like those of LDPE and will biodegrade in industrial composters, is commercially available [14,15]. PBS has properties comparable to those of polypropylene [15], whereas PCL has a relatively low melting temperature of 60 °C [16]. Production rates for these biodegradable polymers are increasing, but most are still manufactured from fossil fuel-based feedstocks. The third option is biodegradable polymers synthesized from bio-based feedstocks (i.e., bioplastics). Polymers produced in high volumes include polylactic acid (PLA), polyhydroxyalkanaotes (PHAs), bio-polybutylene succinate (bio-PBS), and starch blends [13]. These polymers can be degraded in industrial or home composting systems and are now being used in applications such as food packaging [17], compost and plastic bags [18], and agriculture and horticulture films [18,19]. These bioplastics, however, remain more expensive than fossil fuel-based plastics [17,18,19] and have significant limitations regarding their properties [18,20]. 

This work focused on PLA and PHA. PLA is synthesized from lactic acid (2-hydroxypropionic acid), a naturally occurring organic acid that can be produced by chemical synthesis or fermentation [21]. Although PLA has three stereochemical forms, poly(L-lactide) (PLLA), poly(D-lactide) (PDLA), and poly (DL-lactide) (PDLLA) [21], most commercial applications employ PLA copolymers consisting mainly of L-lactide, with small amounts of D-lactide and meso-lactide [13]. The properties of PLA can vary significantly depending on the ratio and distribution of these isomers, as well as the molecular weight of the polymer. Depending on the stereochemistry and thermal history of the material, PLA can be an amorphous or semicrystalline polymer. PLLA and PDLA are considered semicrystalline polymers, while the atactic copolymer, PDLLA, is amorphous. For the semicrystalline PLAs, glass transition temperatures (T_g_) range from 50 °C to 65 °C and the melting temperatures (T_m_) vary from 130 °C to 180 °C, depending on the specific structure; these temperatures play important roles in determining the suitable temperature ranges for various applications [13,22]. With the amorphous PLAs, the glass transition temperature is a critical factor that determines the maximum use temperature in most commercial applications [13]. While PLA has impressive characteristics, its drawbacks include high brittleness, i.e., an elongation at break of less than 10%, low impact strength, poor water barrier properties, high gas permeability, and a relatively low heat distortion temperature [22,23]. 

PHAs have been synthesized by microorganisms using many hydroxyalkanoate building (HB) blocks [24], but only a limited number of PHA copolymers have been commercialized. The homopolyester, poly(3-hydroxybutyrate) (P(3HB)), has thermal and mechanical properties similar to those of PP, slow crystallization, a narrow processing temperature range, and a tendency to creep; therefore, P(3HB) is not preferred for many applications [24]. The copolymer poly(3-hydroxybutyrate-co-3-hydroxyvalerate) (P(3HB-co-3HV)) exhibits reduced crystallinity, decreased stiffness, less brittleness, and increased tensile strength and toughness compared to P(3HB) [24]. Its higher melt strength makes it suitable for processes such as extrusion and blow molding [24]. The properties of the copolymer poly(3-hydroxybutyrate-co-4-hydroxybutyrate) (P(3HB-co-4HB)) vary with composition. If the 4HB content is 5–15%, the copolymer is semicrystalline with lower glass transition and melting temperatures, slower rates of crystallization and lower overall crystallinity, and reduced brittleness and improved flexibility compared to P(3HB) [25,26]. In contrast, when the 4HB content is greater than 30%, the P(3HB-co-4HB) is completely amorphous. Therefore, the material exhibits significant increases in elongation at break but lower moduli and tensile strengths [26]. PHAs can be processed by existing polymer-processing equipment, have received FDA clearance, and can be used in food contact applications [24]. 

PLA has been blended with several biodegradable polymers to improve its properties. When Diaz et al. [27] studied blends of PLA with other polymers for blown film extrusion, they found that PHA, PCL, and PBS enhanced tear resistance and PHA improved heat seal strength. Zhang et al. [28] reported that PLA and PHB formed an immiscible blend, significantly improving the crystallinity and crystallization rate of the PLA. The PLA/PHB blends showed improved mechanical properties and biodegradability compared to PLA. Tri et al. [29] also found that PLA/PHB blends with a wide range of PHB contents were immiscible and that the PHB domains could act as nucleating agent for cold crystallization of PLA. When D-limonene was added to blends of PLA and PHB, the blends were miscible and exhibited improvements in tensile properties and oxygen barrier properties [30]. Burgos et al. [31] melt blended PLA, PHB, and additives (OLA and carvacrol) to obtain active films for food packaging applications. The addition of carvacrol improved the antioxidant activity and antibacterial properties of PLA/PHB films. Moreover, blending PLA with highly crystalline PHB did not affect the biodegradation as the PLA/PHB films disintegrated completely under composting conditions after 17 days.

Recycling of neat PLA has been investigated by reprocessing 100% of the polymer for several heat cycles. Pillin et al. [32] evaluated the impact of reprocessing PLA (MFI: 3–6 g/10 min) during injection molding, whereas Żenkiewicz and coworkers [33] examined the effect of multiple (≤10) twin-screw extruder cycles on PLA (MFI: 5–7 g/10 min). The glass transition and melt temperatures remained relatively constant as the number of extrusion cycles increased [33], but both temperatures decreased with more injection molding cycles [32]. With both processes, the cold crystallization temperature decreased and the level of crystallinity increased with the number of heat histories. Although the tensile properties decreased with an increasing number of processing cycles, the decrease was more pronounced with injection molding. With more heat histories, the Charpy impact strength decreased sharply [33], the melt flow index increased significantly [33], and the rheological properties decreased significantly [32]. This behavior was attributed to chain scission during reprocessing and was confirmed by reductions in molecular weight [32]. Such chain scission is likely greater with high shear processes such as injection molding and it has been observed with a wide range of polymer systems. Żenkiewicz et al. [33] also reported that the rates of water vapor and oxygen transmission steadily increased as the PLA was reprocessed.

There also have been limited studies of recycling for blends of PLA with a second polymer. Zembouai and coworkers [34] evaluated a 50% poly(3-hydroxybutyrate-co-3-hydroxyvalerate) (PHBV)/50% PLA blend using a single-screw extruder, while Farias et al. [35] examined a 70% PLA/30% PHB blend using a twin-screw extruder. As expected, reprocessing reduced the molecular weight of the base polymers and the blends (due to chain scission). The shorter chains had less pronounced effects on the neat PLA and PHBV/PLA blend; the effects were much greater for neat PHBV [34]. With the PLA/PHB blend there were some transesterification reactions, although the blend remained immiscible [35]. Both groups noted the impact of recycling on the crystallization of the PLA.

Although aPHA is not miscible with PLA, it provides significant improvements in PLA’s mechanical properties when added at 10–30 wt. % to PLA [36]. The properties of the blends offer significant potential as bio-based, biodegradable substrates for thermoformed packaging. No studies have evaluated the impact of recycling on PLA/aPHA blends. Given the level of scrap in thermoforming, this work investigated the impact of reprocessing on the processing and properties of neat PLA and two PLA/aPHA blends. The neat PLA and PLA/aPHA blends were subjected to five heat histories in a single-screw extruder. For each heat cycle, the material systems were evaluated for their performance during extrusion and injection molding, the thermal and rheological properties of the extruded compounds, and the tensile and Izod impact properties of molded parts. FTIR was used to evaluate changes in PLA’s chemical structure during processing. These properties will determine whether PLA/aPHA blends can be recycled and, if viable, enable users to determine the amount of scrap PLA/aPHA material that can be added to neat resin to create extruded sheet for thermoformed packaging applications.

## 2. Materials and Methods

### 2.1. Materials

The PLA (NatureWorks, Ingeo Biopolymer 4032D, Plymouth, MN, USA) had a reported melt flow rate of 7 g/10 min (210 °C, 2.16 kg), a melt density of 1080 kg/m^3^ at 230 °C, and a melting point of 155–170 °C [37]. A masterbatch of 55 wt.% PLA and 45 wt. % aPHA was provided by CJ Biomaterials (grade: MA1205P, Woburn, MA, USA). This masterbatch had a reported melt flow rate of 5–8 g/10 min (190 °C, 2.16 kg) and density of 1220 kg/m^3^; the reported glass transition temperatures for the aPHA and PLA used for the masterbatch were about −17 °C and 60 °C, respectively, whereas the reported melt temperature of the PLA was 150 °C to 170 °C. Three different materials—referred to as 100 wt. % PLA, 90 wt. % PLA, and 75 wt. % PLA—were investigated during this work; the compositions of these materials are listed in Table 1. For processing, the PLA, the PLA/aPHA masterbatch, and the extruded materials were dried in a desiccant dryer (Dri-Air Industries, model: MPD-30D, East Windsor, CT, USA) at 75 °C for 4 h.

### 2.2. Extrusion and Molding of the Blends

For each of the three materials in Table 1, a 40 kg batch was prepared for extrusion. The PLA and the masterbatch were weighed and the pellets mixed prior to their transfer to the hopper. The mixture of pellets was gravity fed to a 38 mm single-screw extruder with a square-pitched metering screw having a length-to-diameter ratio of 27:1 and compression ratio of 2:1 (Davis-Standard, model: HPE, Pawcatuck, CT, USA). The melt passed through a breaker plate before being formed with a 4 mm diameter strand die. To reduce degradation of the biopolymers, no screen pack was employed. The extruded strand was cooled using two water baths with a cooling distance of about 3.66 m (which provided sufficient cooling time for the strands to solidify). The solidified strands were then passed through a pelletizer (Bay Plastics Machinery Company LLC, Bay City, MI, USA). For the extrusion trials, the screw speed was 25 RPM, whereas the barrel and die temperatures were set at the suggested temperatures of 180 °C to 200 °C and 190 °C, respectively [37]. For each blend composition, a set of five heat cycles was performed with similar processing conditions. During the extrusion, drive (motor) load was recorded from the controls for the extruder’s drive system, the head pressure was measured using a pressure transducer (Davis Standard, model: 5863606, Pawcatuck, CT, USA), and residence time was determined by timing the travel of color concentrate from the feed port to the die exit. 

Due to difficulties in obtaining stable reciprocating screw plastication of the 100% PLA materials, two injection molding machines and compression molding were used to produce the test specimens. First, a plunger injection molding machine (Xplore micro injection molder, model: IM12, Sittard, The Netherlands) was utilized to fabricate ASTM D638-14 [38] type 5 tensile bars as well as disks for parallel plate rheology. For injection molding of both the tensile bars and disks, the barrel initially was filled with pellets and these pellets were compacted thoroughly using the plunger. Then material was allowed to remain in the barrel for 2 min before being injected into the mold. The temperature of the barrel was maintained at 210 °C, while the mold temperature was set at 65 °C. The combined injection and packing stroke occurred in three steps with step 1 being 3 bar for 3 s, and steps 2 and 3 each being 5 bar for 3 s. Subsequently, the injected samples were promptly removed from the mold and cooled to room temperature. The test bars required for ASTM D256-10 (2018) [39] Izod impact testing of the blends with 90% and 75% PLA were injection molded using a reciprocating screw injection molding machine (Arburg Holding GmbH + Co KG, model: Allrounder 320 C Golden Edition, Loβburg, Germany). The barrel temperature zones gradually increased from 170 °C to 190 °C, the injection velocity was 110 mm/s, the packing was 970 bar for 1 s, the holding was 725 bar for 14 s, the cooling time was 25 s, and the mold temperature was 70 °C. 

A compression press (Carver, model: 4394.4PL3003, Wabash, IN, USA) was employed to produce impact specimens from compounds that were 100% PLA and PLA/aPHA blends with 90% and 75% PLA. To begin the compression molding process, a thin aluminum plate was covered with aluminum foil and the impact bar mold was positioned over the foil. The impact bar molded was then adequately filled with material pellets and another aluminum plate covered with foil was placed over it. Compression molding occurred using a set temperature of 190 °C, compression time of 5 min, and compression force of 245 kN. Cooling of the molded impact bars was performed in a different compression press (Carver, model: 4394.4PL3003, Wabash, IN, USA). For cooling, the set temperature was 25 °C, the compression force was 102 kN, and the cooling time was 5 min. (This two-press system enabled faster compression molding of samples.)

### 2.3. Characterization of the Blends

Thermal analysis was performed using thermogravimetric analysis (TGA) and differential scanning calorimetry (DSC). The samples were not dried prior to these measurements. TGA of the PLA and PLA/aPHA blends was carried out on a Mettler Toledo, model: TGA 2 (SF) machine (Columbus, OH, USA). The samples were heated from 50 °C to 600 °C with a heating rate of 20 °C/min. The measurement was carried out in a nitrogen atmosphere (with a flow rate 70 mL/min). The data were analyzed using STARe software, v.16.40a (Mettler Toledo, Columbus, OH, USA). The critical value was the onset degradation temperature (T_d_), i.e., a weight loss of 5%; the maximum degradation temperatures were also obtained from the DTG curves. The thermal properties, including the melting, crystallization, and miscibility, of the PLA and PLA/aPHA blends were investigated using a differential scanning calorimeter (Mettler Toledo, model: 3+, Columbus, OH, USA). Measurements were performed by heating ~6 to 10 mg samples in an aluminum crucible (covered with an aluminum lid) from 25 °C to 210 °C with a heating rate of 10 °C/min. The specimens were kept at 210 °C for 1 min and then cooled from 210 °C to −40 °C with a cooling rate of 2 °C/min or 20 °C/min. Before the second heat cycle, the samples were kept at −40 °C for 1 min and then heated from −40 °C to 210 °C with a heating rate of 10 °C/min. The measurements were carried out in nitrogen atmosphere (with a flow rate of 20 mL/min). The data were analyzed using STARe software, v.16.40a (Mettler Toledo, Columbus, OH, USA). The critical values were the glass transition and melting temperatures, the crystallization and cold crystallization temperatures, and the crystallinity.

Melt flow rate (melt index) was measured in accordance with ASTM D1238-10 [40] using an extrusion plastometer (Dynisco, model: LMI, Franklin, MA, USA). The barrel temperature was 190 °C and the applied weight was 2.16 kg. Three measurements were made for each sample. Prior to testing, the resin pellets were dried at 45 °C for 24 h in a hot air oven.

A parallel plate rheometer (TA Instruments, model: ARES-G2, New Castle, DE, USA) was used to evaluate the effect of multiple heat cycles on the rheological properties of PLA and the PLA/aPHA blends. The rheometer’s plates had diameters of 25 mm and the gap was 1.5 mm. Prior to testing, the injection molded disks were dried at 45 °C for 24 h in a hot air oven. An initial strain sweep at a fixed frequency determined the linear viscoelastic region for the materials. The frequency sweeps were performed within the frequency range of 0.1 rad/s to 100 rad/s at 190 °C with a strain of 5%. The rheological properties of each sample were conducted two times and mean of the results are reported.

Fourier-transform infrared (FTIR) spectra of the neat PLA, masterbatch, reprocessed PLA, and PLA/aPHA blends were measured using an FTIR spectrometer (Thermo Fisher Scientific, model: Nicolet™ iS50, Waltham, MA, USA) in ATR mode. The room temperature measurements were performed at 4 cm^−1^ resolution and 64 scans.

Tensile properties of the molded specimens were measured using a universal testing machine equipped with a 2 kN load cell (Instron, model 34SC-2, Norwood, MA, USA). The testing was conducted in accordance with ASTM D638-14 and the strain rate was 50 mm/min. Prior to testing, all specimens were conditioned at room temperature (20 °C) for 48 h. The width and thickness of each specimen were measured using a vernier caliper. For each material–heat history combination, five specimens were tested. The measured data collected by the machine’s software (Bluehill Universal, v.4.06, Norwood, MA, USA) were used to determine the tensile modulus, tensile strength at yield and break, and the tensile strain at yield and break.

Notched Izod impact testing was performed in accordance with ASTM D256-10 (2018) using a machine from Testing Machines Incorporated (model: TMI-43, New Castle, DE, USA) and 0.45 and 4.5 kg hammers, depending on the material. The specimens were notched using a notching cutter (Testing Machines Incorporated, model: TMI 22-05, New Castle, DE, USA). The notched samples were conditioned at room temperature (20 °C) for 48 h prior to testing. Ten specimens were tested for each material.

## 3. Results and Discussion

### 3.1. Performance during Extrusion and Injection Molding

For PLA and the PLA/aPHA blends, the pellets fed readily into the extruder and the melts were easily formed into strands. The strands did require a water bath system that was twice the length of a water bath used for materials like PE and PP. This requirement was not unexpected since PLA is slow to crystallize. 

**Figure 1:**
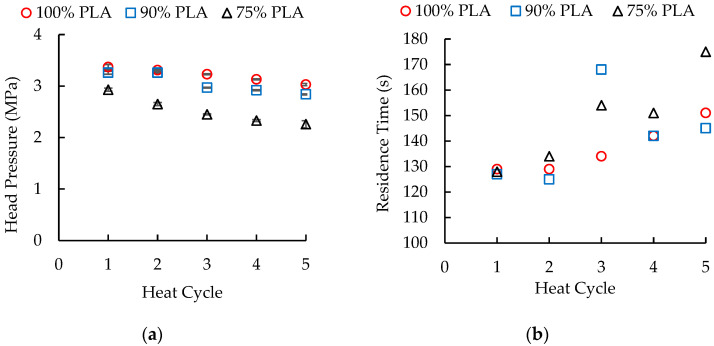
The effect of heat cycles on (**a**) the head pressure and (**b**) the residence time during single-screw extrusion of PLA/aPHA blends with 100, 90, and 75 wt. % PLA.

For each extrusion trial, the head pressure was stable, i.e., the variation was less than 2% of the head pressure. As shown in Figure 1a, the head pressure decreased as larger amounts of aPHA were added to the PLA. For the first heat history, the head pressures were 3.38 MPa, 3.26 MPa, and 2.93 MPa for blends with 100%, 90%, and 75% PLA, respectively. This decrease in head pressure was not unexpected since the PLA/aPHA masterbatch had already undergone one heat history during its mixing. Complex viscosity measurements (discussed later) showed a significant decrease in the viscosity of PLA after one heat history and prior work (unpublished) has shown that the aPHA has a lower viscosity than the PLA. The head pressures also decreased linearly with each additional heat (extrusion) cycle. The rates of decrease were −0.086, −0.118, and −0.166 MPa/heat cycle, respectively, for the materials with 100 wt. %, 90 wt. %, and 75 wt. % PLA; the respective coefficients of determination were 0.99, 0.89, and 0.94. These rates of decrease were consistent with reductions in the molecular weight of the polymer systems; higher molecular weight systems exhibit more resistance to flow. In summary, addition of aPHA to the PLA produced a reduction in the overall head pressure and a greater decrease in the head pressure with each heat history. 

The residence time measurements exhibited unexpected trends (Figure 1b). Typically, the residence time, which represents the duration of material flow within the extruder, increases as the molecular weight, and therefore the viscosity, decreases. This trend was clearly present with 100 wt. % PLA; the residence time increased in a non-linear fashion from 129 s to 151 s over the five heat cycles. In contrast, the blend with 90 wt. % PLA had residence times of about 126 s for heat cycles 1 and 2, 168 s for heat cycle 3, and about 144 s for heat cycles 4 and 5. Decreasing the PLA content to 75 wt. % produced a more gradual increase in residence time from 128 s to 175 s, but a residence time spike was still present for the third heat cycle. This residence time behavior indicated a potential alteration in the rheological properties of the materials.

**Figure 2:**
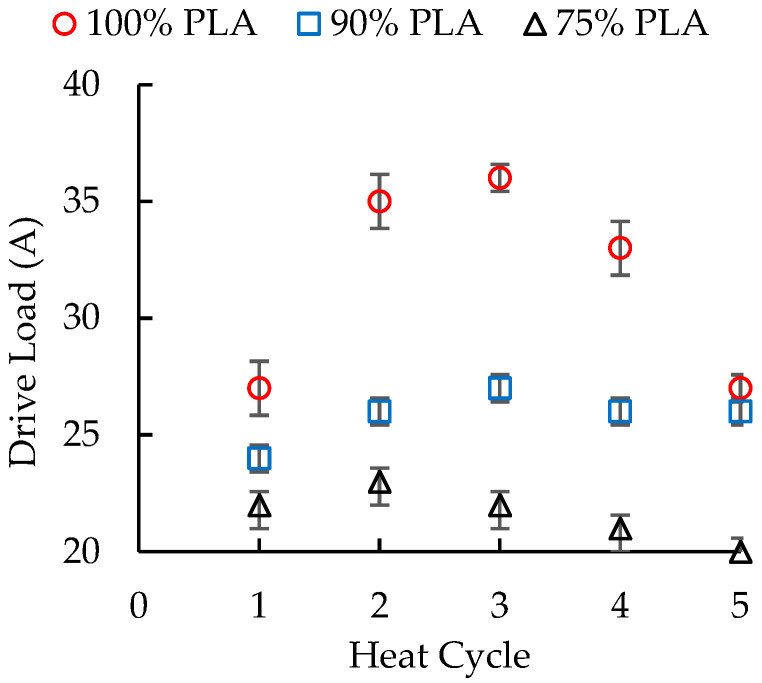
The effect of heat cycles on the drive load during extrusion of PLA/aPHA blends with 100, 90, and 75 wt. % PLA.

The load on the drive motor (drive load) exhibited a very different trend compared to the head pressure (Figure 2). For 100 wt. % PLA, the drive load increased with heat cycles 2 and 3, but then decreased with heat cycles 4 and 5. As a result, the drive load was 27 A for both heat cycle 1 and 5 and 36 A for heat cycle 3. This trend was unexpected because the drive load typically increases as the molecular weight, and therefore the melt viscosity, of the polymer decreases. The lower viscosity melt increases the level of pressure flow (and sometimes leakage flow), creating more work for the extruder’s drive motor. Addition of aPHA to the PLA decreased the overall motor load because these blends had lower viscosities than the 100 wt. % PLA. It also significantly changed the drive load-heat cycle trend. The blend with 90 wt. % PLA exhibited the expected trend, with the drive load gradually increasing from 24 A to 26 A over the first three heat cycles and remaining relatively constant for the last two heat cycles. When the PLA content was 75 wt.%, the drive load showed a similar pattern, but the drive load gradually decreased over the next three heat cycles from 23 A to 20 A. Although these changes in drive load were not reflected in the extrusion of the strands, they did suggest changes in the melt viscosity of the materials.

The factor causing the extruder drive load differences was more pronounced when the blends were plasticated using a reciprocating screw injection molding machine. The PLA/aPHA blends with 90 and 75 wt. % PLA easily molded into test specimens. The 100 wt. % PLA materials, however, exhibited inconsistent plastication, i.e., melting and building a shot of molten polymer in the injection unit. In some molding trials, the drive motor for screw rotation would overtorque, thereby preventing the screw from rotating. At other times, the screw would rotate but not force melt across the non-return valve to build a shot; this problem was called screw slip. Both overtorquing and slip occurred after successful plastication during 10–15 molding cycles. There were no issues with injection of the molten PLA shot into the mold, but once plastication problems started, no further molding could occur. The overtorquing and slip were observed in both a hydraulic machine (Arburg, model: Allrounder 320 C Golden Edition, Loβburg, Germany) and an all-electric machine (Sumitomo, model: SE 75 DUZ, Cleveland, OH, USA). An extensive investigation of plastication parameters did not eliminate this problem, which seemed to be specific to this grade of PLA (NatureWorks, Ingio 4032D, Plymouth, MN, USA); other grades of PLA have been successfully molded using both injection molding machines. 

Limited information is available about this grade of PLA, which is “designed” for higher temperature extrusion. The increases in drive motor suggested increases in melt viscosity. Yoo et al. [41], however, found that the viscosity of this PLA is very sensitive to changes in temperature and exhibits a large decrease in viscosity between 190 °C and 210 °C. Mallet et al. [42] reported low draw and blow-up ratios when using this PLA for blown film extrusion, but the issue seemed to be related to low molecular weight because it was reduced by adding chain extenders to the PLA. In the future, further investigation of this PLA’s performance in injection molded may be warranted. 

### 3.2. Thermogravimetric Analysis

Thermogravimetric analysis (TGA) was performed to determine the onset degradation temperature (T_d_) and the maximum decomposition temperature for the neat PLA, the masterbatch, and the PLA/aPHA blends. The degradation of the neat (unprocessed) PLA occurred in one stage. The unprocessed PLA had a T_d_ of 357.92 °C and a maximum decomposition temperature of 380.00 °C. In contrast, degradation of the PLA/aPHA masterbatch occurred in two stages and the T_d_ was 297.68 °C. Since PLA and aPHA form an immiscible blend, the 55 wt. % PLA and 45 wt. % aPHA in the masterbatch had a co-continuous morphology. Given that PHA degrades at a lower temperature than PLA, the T_d_ value was associated with the aPHA and PLA started to degrade at a higher temperature.

**Figure 3:**
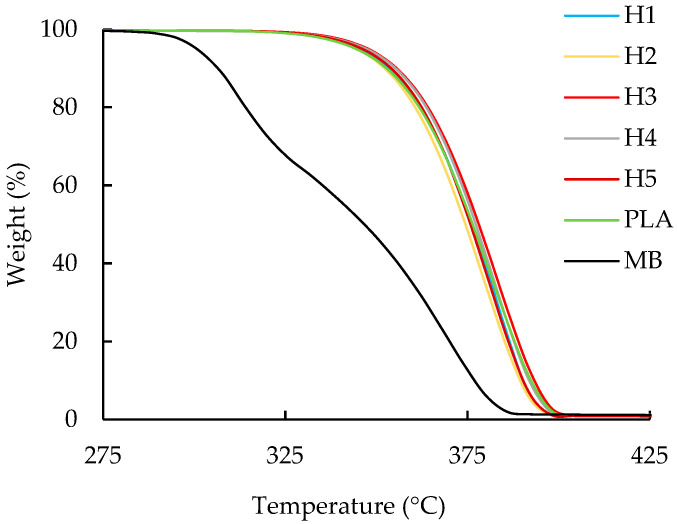
Weight loss vs. temperature curves for TGA analysis of the unprocessed PLA (PLA), the PLA/aPHA masterbatch (MB), and PLA reprocessed in a single-screw extruder; H1 to H5 indicate heat cycles 1 to 5.

As shown in Figure 3, the T_d_ did not change when the blends with 100% PLA were reprocessed in the single-screw extruder; the average T_d_ for the five heat cycles was 358.16 °C ± 1.61 °C. In contrast, when a different PLA was reprocessed in a twin-screw extruder, the T_d_ dropped 4 °C after the first heat cycle and another 3 °C over the next four heat cycles [35]. The difference between that report and the current results may be due to the greater stability of the PLA used for this work. The maximum decomposition temperature was constant, with an average value of 379.1 ± 1.7 °C; these values were similar to the temperature of 380.00 °C obtained for the neat PLA. 

**Figure 4:**
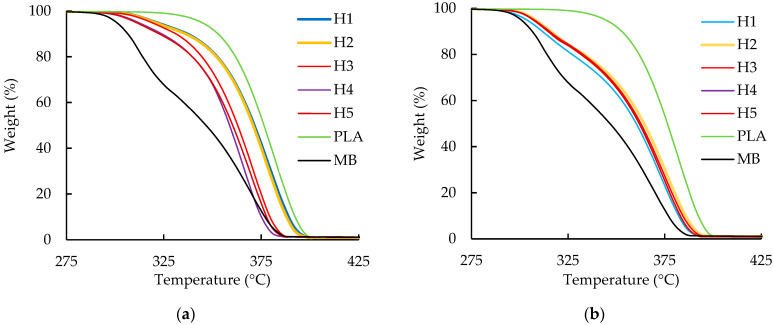
Weight loss vs. temperature curves for TGA analysis of unprocessed PLA (PLA), the PLA/aPHA masterbatch (MD), and PLA/aPHA blends, with (**a**) 90 wt. % PLA and (**b**) 75 wt. % PLA reprocessed in a single-screw extruder; H1 to H5 indicate heat cycles 1 to 5.

The TGA curves for the PLA/a PHA blends are shown in Figure 4. For the PLA/aPHA blend with 90 wt. % PLA, the T_d_ showed no trends. The average value of 334.19 °C ± 2.65 °C was, however, about 22 °C lower than for neat PLA. More importantly, the entire curve shifted to lower temperatures as the number of heat cycles increased (Figure 4a). The shift was similar for heat cycles 1 and 2, somewhat greater for heat cycle 3, and even greater for heat cycles 4 and 5. The maximum decomposition temperatures for the blends with 90 wt.% PLA decreased gradually from 377.0 °C to 366.7 °C. In contrast, the PLA/aPHA blends with 75 wt. % PLA also exhibited T_d_ values that did not vary with heat cycle. The average T_d_ of 300.50 °C ± 1.06 °C was about 60 °C lower than for neat PLA. The weight-temperature curve generally shifted to even lower temperatures, but the curves for all five heat cycles overlapped (Figure 4b). A similar shifting of the weight–temperature curves was observed when a 70/30 blend of PLA and PHB was reprocessed using a twin-screw extruder [35]. For the blends with 75 wt.% PLA, the maximum decomposition temperature was constant, with an average value of 372.2 ± 1.6 °C; due to the addition of aPHA, these values were lower than the temperature of 380.00 °C obtained for the neat PLA. Overall, these patterns for the onset degradation temperature and maximum decomposition temperature suggest changes in the morphology of the two blends.

Overall, the decrease in head pressure (δΔP) observed during extrusion decreased linearly with higher onset degradation temperatures. The correlation can be written as

δ(ΔP)/heat cycle = *m* T_d_ − *b* (1)

where the constants *m* and *b* were, respectively, 0.0014 MPa/°C heat cycle and 0.5702 MPa/heat cycle; the coefficient of determination was 1.00. This behavior suggests that the PLA/aPHA blends are less thermally stable than the PLA and would undergo more rapid decreases in viscosity during reprocessing. 

### 3.3. Differential Scanning Calorimetry

For differential scanning calorimetry (DSC) measurements, the heating rate was 10 °C/min, but the cooling rates were 2 °C/min and 20 °C/min. The slower cooling rate reflected the slow cooling when injection molding the tensile specimens in the plunger injection molding machine and compression molding one set of impact specimens. 

Table 2 presents the T_g_ and T_m_ values obtained from DSC measurements with both cooling rates. When the cooling rate was 2 °C/min, the T_g_ values of PLA were 62 °C in the unprocessed resin and 64 °C in the PLA/aPHA masterbatch. This temperature remained a relatively constant 62 °C in the extruded 100% PLA and was about 65 °C and 66 °C, respectively, for the blends with 90% and 75% PLA. The T_g_ of aPHA, which was about −15 °C in the masterbatch, increased to a steady −19 °C in the extruded PLA/aPHA blends. In contrast, the T_m_ values were 168 °C and about 170 °C, respectively, for the unprocessed PLA and masterbatch. The T_m_ values remained at 169–170 °C for all extruded materials. As expected, the T_g_ and T_m_ values of the unprocessed PLA increased about 2 °C when measured with faster cooling rate of 20 °C/min; in the masterbatch, the T_g_ and T_m_ values of PLA were the same as with the slow cooling rate, but the T_g_ values of aPHA increased by about 5 °C. The T_m_ values of the reprocessed PLA and PLA/aPHA blends remained at 168–171 °C when measured with a cooling rate of 20 °C. PLA’s T_g_ for the reprocessed 100% PLA increased by about 1 °C, whereas it decreased by 3–4 °C in the blends. The T_g_ for aPHA was not measurable for the blend with 90 wt. % PLA, but it increased by about 5 °C when the PLA content was 75 wt. %. Two distinct glass transition temperatures confirmed the immiscibility of the PLA and aPHA. The lack of change in the glass transition and melting temperatures suggests that reprocessing did not change this lack of miscibility.

When PLA was reprocessed using an injection molding machine, the T_g_ decreased about 10 °C and the T_m_ decreased about 4 °C during five heat cycles [32]. In contrast, a different PLA reprocessed in a twin-screw extruder exhibited no significant change in T_g_ and T_m_ [33]. These results along with the data from this work reflect the differences in the stability of the PLA materials and the shear heating produced in the processes. With this work, the PLA did not undergo sufficient chain scission to affect the transition temperatures, which typically depend on intermolecular attractions (rather than molecular weight).

**Figure 5:**
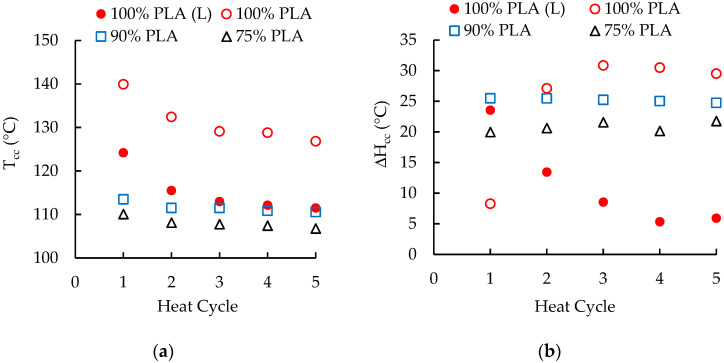
The effect of heat cycles on the (**a**) cold crystallization temperatures and (**b**) cold crystallization enthalpy of PLA/aPHA blends with 100, 90, and 75 wt. % PLA; (L) indicates a cooling rate of 2 °C/min, whereas the other values were obtained with a cooling rate of 20 °C/min.

Cold crystallization (recrystallization) was observed with the neat and reprocessed 100 wt. % PLA. This behavior was not unexpected since PLA is slow to crystallize and, when PLA is reheated to above its T_g_, greater mobility of the polymer chains enables crystallization before melting. Cold crystallization has been reported for DSC cooling rates of 3, 10, and 20 °C/min [33,34,35]. With a cooling rate of 2 °C/min, the cold crystallization temperature (T_cc_) for the neat PLA was about 135 °C. This temperature dropped to about 124 °C after one pass through the extruder and with additional processing in the extruder, the T_cc_ decreased slowly from 116 °C to 112 °C (Figure 5a). When the cooling rate was 20 °C/min, the neat PLA exhibited no T_cc_, but the reprocessed PLA had a similar pattern of T_cc_ values, which were 140 °C after the first heat cycle and decreased to 127 °C after the fifth heat cycle (Figure 5a). The cold crystallization enthalpy (ΔH_cc_) values for PLA, however, exhibited very different trends. At a heating rate of 2 °C per minute, the ΔH_cc_ for the unprocessed PLA was 16.87 J/g and the enthalpy ΔH_cc_ values for the reprocessed 100 wt. % PLA decreased from 23.55 J/g to 5.9 J/g over the five heat cycles (Figure 5b). Similar results were reported for a different PLA reprocessed using a twin-screw extruder; the T_cc_ decreased 5 °C during five heat cycles [35]. Increasing the heating rate to 20 °C/min produced no ΔH_cc_ value for the unprocessed PLA, a relatively low ΔH_cc_ (8.27 J/g) after one heat cycle, and much higher ΔH_cc_ values (~30 J/g) with additional reprocessing (Figure 5b). The changes in ΔH_cc_ reflected shortening of the polymer chains that make crystallization and recrystallization easier. The heating rates determined whether cold crystallization will be dominant.

With the slow cooling rate (2 °C/min), cold crystallization did not occur with the masterbatch and the PLA/aPHA blends having 90 and 75 wt. % PLA. In contrast, the masterbatch and the two blends underwent cold crystallization when the cooling rate was 20 °C/min. At this cooling rate, the T_cc_ was 0 °C for the neat PLA and 114 °C for the PLA/aPHA masterbatch. As shown in Figure 5a, T_cc_ for the blends with 90 and 75 wt. % PLA decreased slightly with each additional heat cycle, and were lower for the for the blend with 75% PLA. The respective average T_cc_ values were 112 °C and 108 °C. The averaged ΔH_cc_ values were 0, 15, 25, and 21 J/g for the neat PLA, PLA/aPHA masterbatch, PLA/aPHA blend with 90 wt. % PLA, and the PLA/aPHA blend with 75 wt. % PLA, respectively. Limited change in the ΔH_cc_ occurred when the PLA content was 90 wt. %, but the ΔH_cc_ for the blend with 75 wt. % PLA increased from 20 J/g to 22 J/g with more reprocessing (Figure 5b). These changes reflected the greater mobility of the polymer chains in the PLA/aPHA blends.

Table 3 summarizes crystallization temperatures (T_c_), crystallization enthalpies (H_c_), and melting enthalpies (H_m_). When the cooling rate was 20 °C/min, the neat PLA, masterbatch, and reprocessed materials exhibited no crystallization temperatures and crystallization enthalpies. In contrast, these values were present when the cooling rate was 2 °C/min. With the neat PLA and the blend with 100 wt. % PLA after one heat cycle, there was no clear crystallization temperature. During heat cycles 2 to 5, the crystallization temperature increased from about 98 °C to 100 °C. Similar behavior previously has been reported. For example, PLA reprocessed with a twin-screw extruder exhibited a similar lack of crystallization temperature for the neat PLA and a 5 °C increase in crystallization temperature over five heat cycles [35]. For the PLA/aPHA blends having 90 wt. % and 75 wt. % PLA, the crystallization temperatures were about 102–103 °C and 104–105 °C, respectively, and did not change significantly over the five heat cycles. With the slower cooling rate, H_m_ was not present for neat PLA and was 15.87 J/g for the PLA/aPHA masterbatch. With the reprocessed PLA, H_m_ increased from not present after heat cycle 1 to 16.17 J/g after heat cycle 5. The PLA/aPHA blends with 90 wt. % and 75 wt. % PLA had relatively constant H_m_ values of about 26.64 and 21.27 J/g, respectively. 

**Figure 6:**
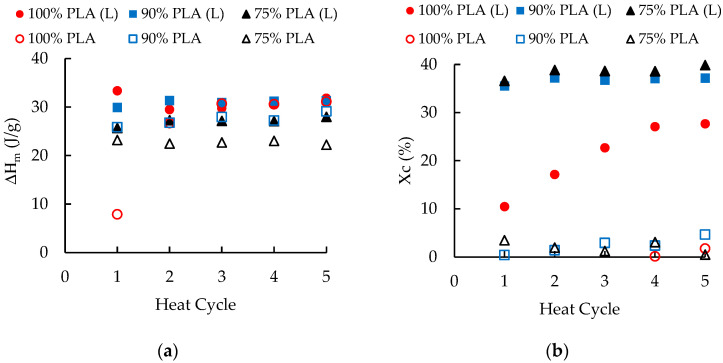
The effect of heat cycles on the (**a**) melting enthalpy and (**b**) degree of crystallinity for PLA and PLA/aPHA blends with 90 wt. % PLA and 75 wt. % PLA; (L) indicates a cooling rate of 2 °C/min whereas the other results are for a cooling rate of 20 °C/min.

Figure 6 presents the melting enthalpy and the degree of crystallization obtained from DSC measurements. The degree of crystallinity was calculated using [43]:

(2) $$X_{c}=\left[\frac{{∆H}_{m}-{∆H}_{cc}}{{∆H}_{m}^{c}}\right]\times \frac{1}{{wt\%}_{Polymer}}$$

where ΔH_cc_, ΔH_m_, and ${∆H}_{m}^{c}$ are the cold crystallization enthalpy, melting enthalpy, and melting enthalpy of pure crystalline polymer, respectively, and wt%_polymer_ is the weight fraction of the relevant polymer in the blend. The melting heat of pure PLA is 93.6 J/g [43]. With a cooling rate of 2 °C/min, the melting enthalpy was relatively constant for the reprocessed materials (Figure 6a). The ΔH_m_ values were about 18 J/g for neat PLA, 20 J/g for the masterbatch, 31 J/g for the blends with 100 and 90 wt. % PLA, and 27 J/g for the blend with 75 wt.% PLA. In contrast, for the higher cooling rate of 20 °C/min, the melting enthalpy was about 2 J/g for neat PLA and 17 J/g for the PLA/aPHA masterbatch. As shown in Figure 6a, ΔH_m_ values increased from 7 J/g to 30 J/g for the blends with 100 wt. % PLA, increased from 26–29 J/g for the 90 wt.% PLA blend, and remained a steady 23 J/g for the blend with 75 wt. % PLA.

As shown in Figure 6b, the overall result was that samples cooled at 20 °C/min had crystallinity values of less than 3%, whereas the values for the slowly cooled samples were much greater. The degree of crystallinity for samples cooled at 2 °C/min increased with additional heat cycles. Over the first four heat cycles, the degree of crystallinity increased from 10% to 27% in the blend with 100 wt. % PLA; a smaller increase in the degree of crystallinity (from 27 to 28%) occurred for heat cycles 4 to 5. Similar patterns and degrees of crystallinity have been reported for PLA reprocessed with an injection molding machine [32] and a twin-screw extruder [33]. This behavior suggests that reductions in the length of the polymer chains provide the chain mobility needed for great crystallization of PLA. In contrast, the degree of crystallinity for the PLA/aPHA blends was significantly higher (i.e., 35–40%) and did not increase substantially as the blends were reprocessed. The greater degree of crystallinity reflects the greater mobility imparted by the aPHA; this mobility meant that reductions in polymer chain length had a more-limited effect on crystallization. The amorphous nature of the aPHA may affect this pattern. A blend of 50 wt. % PLA and 50 wt. % PHB—where both the PLA and PHB crystallized—exhibited a degree of crystallization pattern like that of the 100 wt. % PLA and the degree of crystallinity increased from about 9% to 18% over five heat cycles [35].

### 3.4. Melt Index

**Figure 7:**
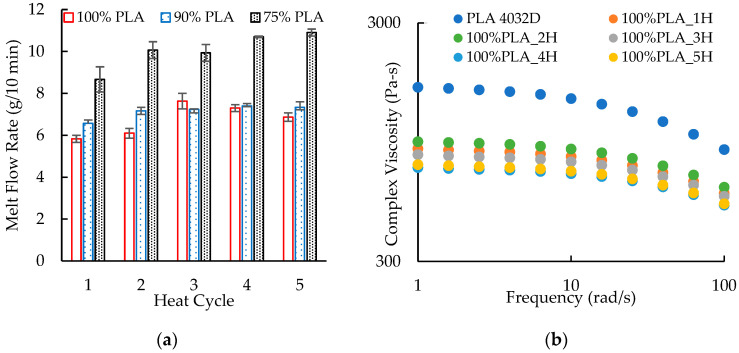
The effect of heat cycle on (**a**) the melt flow rate of PLA/aPHA blends with 100, 90, and 75 wt. % PLA and (**b**) the complex viscosity–frequency curves for neat PLA and reprocessed 100 wt. % PLA.

Figure 7a presents the melt flow rates for the reprocessed PLA. The unprocessed PLA had a melt flow rate of 6.00 ± 0.09 g/10 min. Overall, the heat cycles did not produce major changes in the melt flow rate, but the pattern of the flow rates was interesting. After the first two heat cycles, the melt flow rates of 5.83 and 6.10 g/10 min were similar to the melt flow rate of the unprocessed PLA. The melt flow rate increased to 7.66 and 7.30 g/10 min for heat cycles 3 and 4, respectively, and then decreased to 6.89 g/10 min for heat cycle 5. Chain scission during reprocessing typically produces a continuous increase in melt flow rate. The pattern of melt flow rates for this PLA, however, was consistent with the drive loads observed during extrusion (Figure 2). The high flow rates (and lower melt viscosities) for cycles 3 and 4 would have produced pressure flow and, therefore, greater drive loads.

The masterbatch had a melt flow rate of 11.17 ± 0.05 g/10 min. Combining this masterbatch with the neat PLA to create a blend with 90 wt. % PLA produced a small and sometimes significant increase in melt flow rate compared to the reprocessed PLA (Figure 7a). The melt flow rates generally increased with heat cycle, giving 6.57 g/10 min for heat cycle 1 and 7.33 g/10 min for heat cycle 5. The PLA/aPHA blend with 75 wt. % PLA exhibited the same trend, but the melt flow rates were higher. They varied from 8.67 g/10 min for heat cycle 1 and 10.90 g/10 min for heat cycle 5. 

Since chain scission reduces melt viscosity, melt flow rate typically increases as materials are reprocessed. Most results from this work are consistent with those from prior studies. With reprocessing, PLA had a gradual increase in melt flow rate from 3 g/10 min to 5 g/10 min [33], whereas a blend of 70% PLA with 30% PHB showed a linear increase in melt flow from 9 g/10 min to 33 g/10 min [35]. Both reductions in chain length and changes in the morphology of the blends’ minor phase increased the mobility of the polymer system, thereby increasing its melt flow rate.

### 3.5. Parallel Plate Rheology

Figure 7b presents the complex viscosity–frequency curves for 100% PLA. For neat PLA, the complex viscosity at 1 rad/s (η_1_) was about 1612 Pa-s. Viscosity decreased substantially to 850 Pa-s after the first heat cycle and then less significantly to 840 Pa-s and 765 Pa-s after the third and fifth heat cycles, respectively. Pillin et al. [32] observed a similar reduction in the zero-shear viscosity (η_0_) when PLA was reprocessed using an injection molding machine; η_0_ was 3960 for the unprocessed PLA, 713 Pa-s after one heat cycle, and less than 50 Pa-s after five heat cycles. Zembouai et al. [34] did not provide the viscosity of the unprocessed PLA, but they reported that η_1_ decreased from 2290 Pa-s to 1160 Pa-s during six heat cycles. In addition to the decrease in viscosity, the degree of shear thinning, which is indicated by the slope in the power law region of the complex viscosity–frequency curves, also decreased with reprocessing. This behavior was not unexpected because the shorter polymer chains reduce the resistance to flow; therefore, their alignment in the direction of flow does not have as great an effect on viscosity as would occur with more viscous melts. Neither this work nor data reported by Zembouai et al. [34] showed significant extension of the lower Newtonian plateau (i.e., the region in which complex viscosity does not change with frequency) to higher shear rates. Such extensions of the lower Newtonian plateau would require much great reductions in molecular weight. One unexpected result was the greater viscosity (~900 Pa-s) after the second heat cycle; this result was repeatable and may have been due to changes in the molecular weight of the PLA. 

For the 100% PLA materials, the weight-average molecular weight (M_w_) was calculated from the zero-shear viscosity (η_0_) using [44]:

(3) $$\eta_{0}=K{M_{w}}^{a}$$

where K is 5.5 × 10^−15^ and a is 3.4 for PLA. The calculated molecular weights are listed in Table 4. The first extrusion cycle produced a 16% reduction in the molecular weight of the PLA, whereas additional extrusion cycles produced smaller changes in molecular weight. This reduction in molecular weight would have reduced the melt viscosity to produce the increasing lower head pressures shown in Figure 1a and the longer residence times observed in Figure 1b. The reduction in molecular weight contributed to the lower viscosities and high melt indices of the PLA/aPHA blends since the masterbatch underwent one heat cycle during compounding. The higher molecular weight for heat cycle 2 suggests that the higher drive load in cycle 3 (Figure 2) and the lower melt index for cycle 2 were due to increases in the molecular weight of the PLA (the sample for heat cycle 2 underwent an additional heating cycle to produce the samples for parallel plate rheology and during melt index measurements). In contrast, the variations in molecular weight for heat cycles 4 and 5 are consistent with variations during the rheology measurements. The cause of the increase in molecular weight was not determined during this work. 

**Figure 8:**
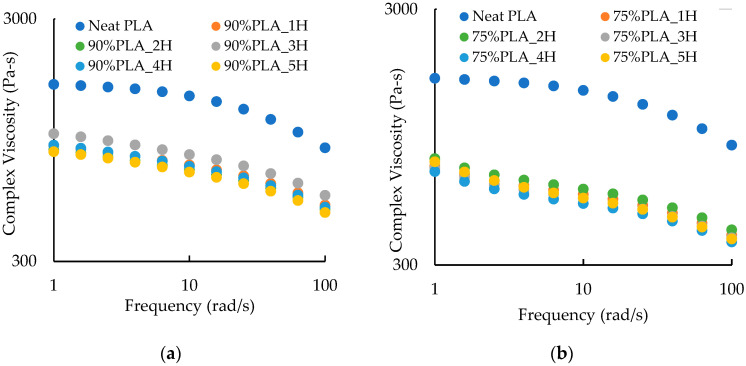
The effect of heat cycle on the complex viscosity-frequency curves for PLA/aPHA blends with (**a**) 90 wt. % PLA and (**b**) 75 wt. % PLA.

As illustrated in Figure 8a, the addition of 10 wt.% aPHA to PLA produced slightly higher complex viscosities at 1 rad/s (h_1_) compared to the reprocessed 100 wt. % PLA. The η_1_ values for the PLA/aPHA blend with 90% PLA were about 900 Pa-s after one heat cycle and gradually decreased to 850 Pa-s after five heat cycles. Curves for these PHA/aPHA blends did not exhibit a lower Newtonian plateau (which was present with the neat and reprocessed PLA). The level of shear thinning also was not clear because the power law region seemed to have shifted higher shear rates. In addition, the viscosity after the third heat cycle was higher (1008 Pa-s). Although this increase in viscosity with the third heat cycle was not expected, it did correspond to a spike in extruder residence time (Figure 1b).

When the PLA content in the PLA/aPHA blends was reduced to 70 wt. %, the reprocessed blends exhibited similar viscosities, with η_1_ being about 740 Pa-s (Figure 8b). The curves also exhibited increases in low viscosity at low frequencies, which was due to the rheological characteristics of the masterbatch. Similar curves were reported for blend of PLA and PHBV [34].

### 3.6. FTIR

**Figure 9:**
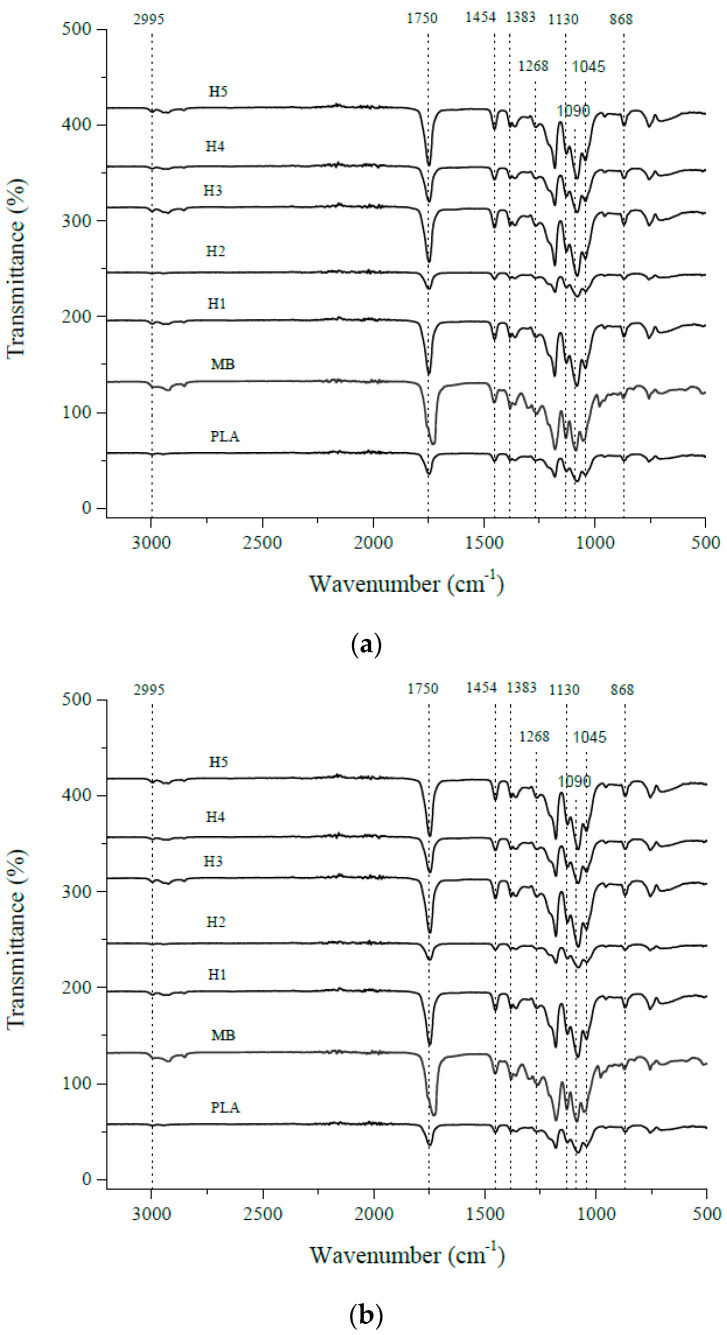

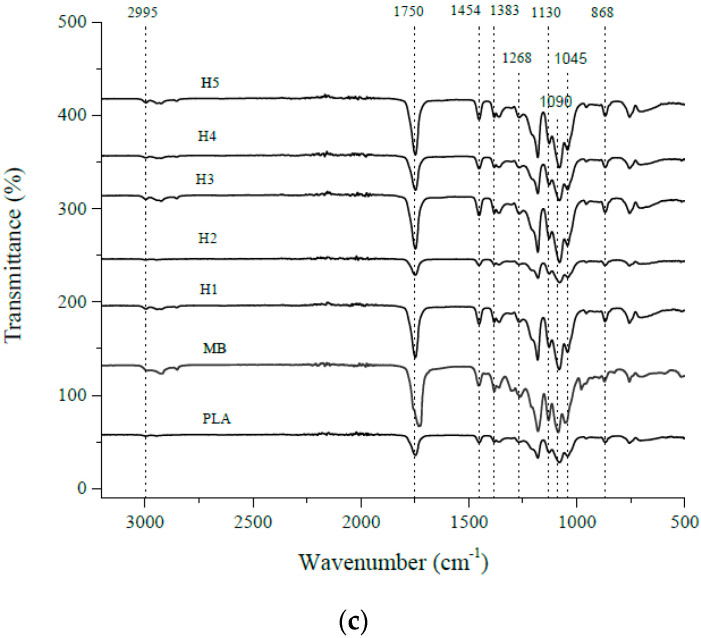
The effect of heat cycle on the FTIR spectra of (**a**) PLA, (**b**) the 90/10 PLA/aPHA blend, and (**c**) the 75/25 PLA/aPHA blend; PLA is the unprocessed PLA, MB is the masterbatch, and H1 to H5 are heat cycles 1 to 5.

Fourier Transform Infrared Spectroscopy (FTIR) was employed to investigate the impact of repeated thermal cycles on chemical and structural alterations. All spectra exhibited distinctive absorption peaks corresponding to functional groups present in PLA (Figure 9). These peaks include the stretching vibration of methyl groups (νasCH3) at 2995 cm^−1^, the symmetric stretching vibration of methyl groups (νsCH3) at 2945 cm^−1^, the stretching vibration of carbonyl groups (νC = O) at 1755 cm^−1^, the asymmetric deformation of methyl groups (δasCH3) at 1452 cm^−1^, the symmetric deformation of methyl groups (δsCH3) at 1383 cm^−1^, the deformation of the first carbon–hydrogen bond (δ1CH) at 1360 cm^−1^, the deformation of carbon–hydrogen and carbon–oxygen bonds (δCH and νCOC) at 1268 cm^−1^, the asymmetric stretching of carbon–oxygen–carbon and the symmetric stretching of methyl groups (νasCOC and rasCH3) at 1212–1185 cm^−1^, the symmetric stretching of methyl groups (rasCH3) at 1130 cm^−1^, the symmetric stretching of carbon–oxygen–carbon bonds (νsCOC) at 1090 cm^−1^, the stretching vibration of carbon–methyl bonds (νC-CH3) at 1045 cm^−1^, the rocking vibration of methyl groups and the stretching vibration of carbon–carbon bonds (rCH3 and νCC) at 956–920 cm^−1^, and the stretching vibration of carbon–carbon–oxygen bonds (νC-COO) at 868 cm^−1^ [45].

As shown in Figure 9a, no substantial changes in absorption peaks were observed between the neat PLA and the reprocessed PLA samples. There were differences in the levels of absorption, particularly for the stretching vibration of carbonyl groups (νC = O) at 1755 cm^−1^, which may be due to the overall heat history (i.e., extrusion, injection molding, and three steps of drying) or changes in the degradation of this PLA material. Figure 9b presents the FTIR results for the PLA/aPHA blends with 90 wt. % PLA; similar results were obtained for the PLA/aPHA blends with 75 wt. % PLA. With the blends, there also were no shifts in the critical PLA absorbance peaks. This observation suggests that, despite undergoing multiple thermal cycles, the chemical and structural properties of the polymer remain largely unchanged. Moreover, the findings imply that different mechanical recycling processes have a limited influence on these structural modifications, indicating the potential applicability of recycled PLA in various fields.

### 3.7. Mechanical Properties of Blends

**Figure 10:**
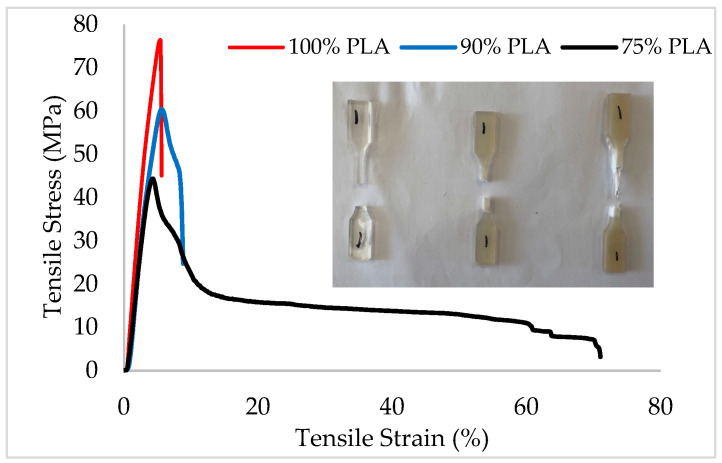
Typical tensile stress–strain curves for PLA/aPHA blends with 100, 90, and 75 wt. % PLA; the inset presents typical broken tensile specimens.

For viable recycling of PLA and aPHA in packaging applications, the mechanical properties must remain stable through multiple processing cycles. Figure 10 presents typical tensile stress–strain curves for the PLA/aPHA compounds with 100, 90, and 75 wt. % PLA, along with images of the typical broken samples. Reprocessed PLA was very brittle and exhibited no clear yield point. The addition of 10 wt. % aPHA to the PLA produced a clear yield point, a decrease in the tensile strength, and a slight increase in the tensile strain or elongation. With 25 wt. % aPHA in the blend, the yield point was present, the tensile strength decreased further, and the tensile strain showed a 14-fold increase compared to the blends with 10 wt. % aPHA. Therefore, analyses were performed for the tensile modulus, ultimate tensile strength, and tensile strain at break.

**Figure 11:**
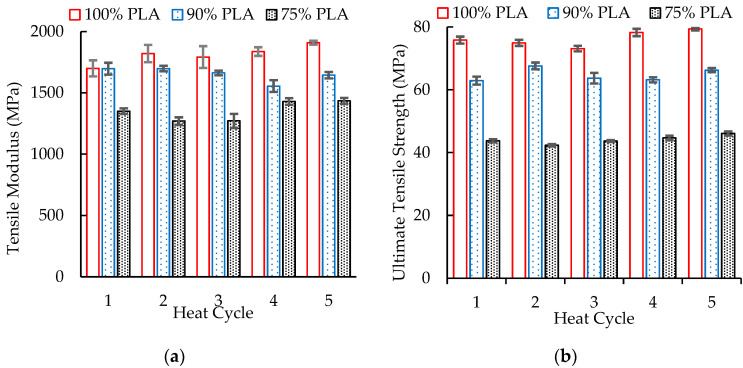
The effect of heat history on the (**a**) tensile modulus and (**b**) ultimate tensile strength of PLA and PLA/aPHA blends.

As shown in Figure 11b, multiple extrusion cycles had little effect on the ultimate tensile strength of the PLA and blends of PLA and aPHA. This behavior was not unexpected since tensile strength depends on interchain attractions rather than the length of the polymer chains; one would not expect significant reductions in tensile strength to occur until the polymers had undergone high levels of chain scission. Previous studies have reported similar findings regarding the influence of multiple heat cycles on the tensile strength of PLA and its blends [33,34,35]. Since aPHA is more flexible than PLA, the PLA/aPHA blends exhibited lower ultimate tensile strengths. With 100, 90, and 75 wt. % PLA, the ultimate tensile strengths were about 73–79 MPa, 63–70 MPa, and 42–46 MPa, respectively. The changes in crystallinity in the DSC may not have been reflected in the tensile specimens because they were cooled at a different rate after injection molding.

The tensile strain at break was not clearly impacted by reprocessing (Table 5). As expected, the tensile strain at break values had larger standard deviations. Although addition of aPHA significantly increased the tensile strain at break, it also increased the variation in the values. The immiscibility of the PLA and aPHA contributed to these changes. Tensile strain at break can depend on the length of the polymer chains. Pillin et al. [32] found that the strain at break of PLA decreased as the number of injection molding cycles increased. Zembouai and coworkers [34], however, reported no significant change in strain at break when reprocessing PLA, PHBV, and PLA/PHVB blends with a single-screw extruder. In general, extruders apply less stress to the polymer chains than injection molding machines, and the resins employed for extrusion typically have longer chain lengths.

**Figure 12:**
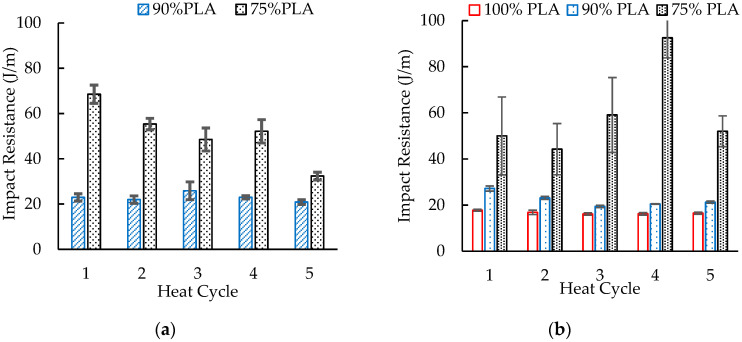
The effect of heat history on the notched Izod impact resistance of (**a**) injection molded and (**b**) compression molded blends of PLA and aPHA.

Figure 12a presents the effect of heat history on the notched Izod impact resistance of the injection molded blends. No data are reported for 100 wt. % PLA because that material could not be injection molded. The blends with 90 wt. % PLA exhibited no significant change in impact resistance with increasing heat cycles. The impact resistance remained at about 23 J/m. In contrast, the impact resistance of blends with 75 wt. % PLA generally decreased with more heat cycles. The overall impact resistance values of 69 J/m to 32 J/m were greater than those of the blends with 90 wt. % PLA. This PLA/aPHA blend was not miscible. Therefore, a higher aPHA content provided greater impact resistance, probably due to the lower crystallinity in those blends and the change in the morphology of the aPHA within the blends. With 10 wt. % aPHA, the aPHA domains were smaller and the impact strength was less sensitive to the degradation of the aPHA. The higher level of aPHA, however, made the impact resistance more sensitive to the decrease in molecular weight that occurred with reprocessing. When the aPHA content was 25 wt. %, domains were larger and may not have been spherical. These findings correlate with changes in melt flow rate shown in Figure 7a.

As illustrated in Figure 12b, the pattern for the notched Izod impact resistance was similar to that with the compression molded test specimens. Over the five heat histories, the 100% PLA provided a relatively constant impact resistance of about 17 J/m, which was consistent with the 16 J/m reported for this grade of PLA [37]. With the blend containing 90% PLA, the impact resistance was greater, but it was a little more sensitive to heat history. The impact resistance was about 27 J/m after one pass through the extruder and dropped to about 21 J/m after cycles 2 to 5. These specimens exhibited complete breaks. In contrast, the blends with 75% PLA primarily showed hinge breaks; therefore, the impact resistance values were calculated from the few samples that exhibited complete breaks. The result was a significantly greater impact resistance of about 60 J/m, but the errors in the measurements did not provide a clear trend for the effects of heat history on this impact resistance.

These results suggest that material composition has a greater effect on impact strength than heat history. As the number of heat cycles increased, Zembouai et al. [34] observed a reduction in the impact strength of PLA/PHBV blends. In contrast, the impact strength of the base polymers (PLA and PHBV) was lower but did not change significantly with reprocessing. Farias et al. [35] also reported that reprocessing produced no significant change in the impact strength of blends of PLA with 30% PHB. 

## 4. Conclusions

Immiscible polylactic acid (PLA)/amorphous polyhydroxyalkanoate (aPHA) blends with 90 and 75 wt. % PLA and a 100 wt. % PLA control underwent five cycles in a single-screw extruder. The PLA and PLA/aPHA blends extruded easily and did not exhibit a critical loss in melt strength, and the melt flow rates and complex viscosities of the blends did not change significantly after the first or second heat cycle. All reprocessed blends exhibited TGA onset of decomposition temperatures that were lower than for neat PLA. The glass transition and melting temperatures were not impacted by reprocessing, but the chain scission associated with reprocessing provided the additional mobility needed for crystallinity to increase with a greater number of heat cycles. Fourier Transform Infrared Spectroscopy indicated no major changes in the structure of PLA for the reprocessed PLA and PLA/aPHA blends. While the tensile properties were not affected by reprocessing, the notched Izod impact resistance of the blend with 75 wt. % PLA decreased by about 50% after five reprocessing cycles. Overall, the PLA/aPHA blends with 25 wt. % aPHA provided good extrudability, great improvements in elongation at break (<100%) and improved impact resistance, and limited sensitivity of these properties to reprocessing, suggesting that the blend could be employed for thermoformed packaging.

## Figures and Tables

**Table 1:** Composition of materials investigated.

Material	100% PLA	90% PLA	75% PLA
PLA (wt. %)	100	90	75
aPHA (wt. %)	0	10	25

**Table 2:** Glass transition and melt temperatures obtained from DSC.

		Cooling Rate = 2 °C/min			Cooling Rate = 20 °C/min		
Material	Heat Cycles	T_g, PLA_ (°C)	T_g, aPHA_ (°C) *	T_m, PLA_ (°C)	T_g, PLA_ (°C)	T_g, aPHA_ (°C) *	T_m, PLA_ (°C)
Neat PLA	0	62	---	168	64	---	170
Masterbatch	0	64	−15	170	63	−11	170
100 wt. % PLA	1–5	62	---	169	63–64	---	168–171
90 wt. % PLA	1–5	65	−19	170	62	---	169–170
75 wt. % PLA	1–5	66	−19	170	61	−14	169

* T_g_ for aPHA could not always be determined from DSC data.

**Table 3:** Summary of crystallization temperatures and enthalpies and melting enthalpies.

		Cooling Rate = 2 °C/min			Cooling Rate = 20 °C/min		
Material	Heat Cycles	T_c_ (°C)	dH_c_ (J/g)	dH_m_ (J/g)	T_c_ (°C)	dH_c_ (J/g)	dH_m_ (J/g)
Neat PLA	0	---	---	17.99	---	---	1.96
Masterbatch	0	106	15.87	20.09	---	---	16.89
100 wt. % PLA	1	---	---	33.36	---	---	7.88
100 wt. % PLA	2–5	98–100	9.19–16.17	30.45	---	---	29.78
90 wt. % PLA	1–5	102–103	23.64	30.95	---	---	27.39
75 wt. % PLA	1–5	104–105	21.27	27.03	---	---	22.73

**Table 4:** Effect of heat cycle on the molecular weights of 100% PLA.

Heat Cycle	0	1	2	3	4	5
η_0_ (Pa-s)	1611	891	953	841	742	764
*M_w_* (g/mol)	137,188	115,229	117,557	113,284	109,209	110,131

**Table 5:** Effect of reprocessing on the tensile strain at break (%).

Heat Cycle	100% PLA	90% PLA	75% PLA
1	5.66 ± 0.28	11.43 ± 7.79	121.8 ± 38.3
2	6.31 ± 0.96	10.14 ± 4.97	99.2 ± 26.6
3	7.01 ± 1.43	6.84 ± 1.74	103.9 ± 57.7
4	6.50 ± 0.26	11.32 ± 5.45	142.9 ± 47.2
5	6.57 ± 0.26	9.90 ± 5.20	158.3 ± 11.5

## Data Availability

The raw data supporting the conclusions of this article will be made available by the authors on request.
